# Identifying candidate diagnostic markers for early stage of non-small cell lung cancer

**DOI:** 10.1371/journal.pone.0225080

**Published:** 2019-11-14

**Authors:** Zhen Wu, Xu Zhang, Zhihui He, Liyun Hou

**Affiliations:** 1 School of Mathematics and Statistics, Southwest University, Chongqing 400715, China; 2 Department of Pediatric Respiration, Chongqing Ninth People’s Hospital, Chongqing 400700, China; University of South Alabama Mitchell Cancer Institute, UNITED STATES

## Abstract

We performed a series of bioinformatics analysis on a set of important gene expression data with 76 samples in early stage of non-small cell lung cancer, including 40 adenocarcinoma samples, 16 squamous cell carcinoma samples and 20 normal samples. In order to identify the specific markers for diagnosis, we compared the two subtypes with the normal samples respectively to determine the gene expression characteristics. Through the multi-dimensional scaling classification, we found that the samples were clustered well according to the disease cases. Based on the classification results and using empirical Bayes moderation and treat method, 486 important genes associated with the disease were identified. We constructed gene functions and gene pathways to verify our result and explain the pathogenicity factor and process. We generated a protein-protein interaction network based on the mutual interaction between the selected genes and found that the top thirteen hub genes were highly associated with lung cancer or some other cancers including five newly found genes through our method. The results of this study indicated that contrast on the gene expression between different subtypes and normal samples provides important information for the detection of non-small cell lung cancer and helps exploration of the disease pathogenesis.

## Introduction

Lung cancer is the most common malignant tumors, which poses a major threat to public health. In 2018, it was predicted that 1,735,350 new cancer cases and 609,640 cancer deaths will occur in the United States, including 13.49% lung cancer cases and 25.27% lung cancer deaths [1]. Lung cancer is the cancer with the highest mortality. There are two major subtypes of lung cancer, small cell lung cancer (SCLC) and non-small cell lung cancer (NSCLC). NSCLC, including two major histopathological subtypes, adenocarcinoma (AC) and squamous cell carcinoma (SCC), accounts for 80% of all lung cancer cases [2]. At present, the most effective treatment for NSCLC is surgical treatment in the early stage and radiotherapy and chemotherapy in the middle and late stage. About 75% of the patients are diagnosed in the middle and late stage. Regardless of the treatment options, the overall survival rate is still very poor [3].

In the recent years, researchers have paid more attention to the mechanism of the occurrence and growth of NSCLC, which has brought new breakthroughs to the diagnosis and treatment of this disease. However, because of the high cost of treatment and the presence of drug resistance, effective treatment is only applicable to a narrow population.

With the development of information technology, using gene expression data resources to solve medical problems has become a general trend. Data mining technology is helpful to extract potential and valuable information related to diseases, so as to effectively prevent and control the diseases. Therefore, gene expression profile analysis has been widely used to identify new potential biomarkers of cancer [4, 5], among which tumor-associated genetic alterations have played essential roles in the tumorigenesis and progression of cancer [6].

In this study, we focus on a particular set of gene expression data associated with early stage of NSCLC. We are interested in this data set of 76 samples because the data set contains detailed information about AC, SCC and normal samples. This information, as our study will show, is critical for the extraction of candidate diagnostic markers for NSCLC. We will use the affy package to read raw data, the edgeR package [7] to filter and normalize the data and the limma package [8] to assess differential expressed genes (DEGs) and perform exploration analysis of the results. Using a multi-dimensional scaling analysis, we will observe the significantly different gene expressions between different NSCLC subtypes and health cases. Applying the linear models in limma package and empirical Bayes moderation in Bioconductor, we will discover more host genes associated with NSCLC. To verify these genes from the underlying biology mechanism, we will use the database for annotation, visualization and integrated discovery (DAVID) [9] to perform the gene ontology (GO) functional analysis [10] and the Kyoto Encyclopedia of Genes and Genomes (KEGG) pathway analyses [11]. Further, the protein-protein interaction (PPI) network will be constructed by search tool for the retrieval of interacting genes/proteins (STRING) database [12], and the Cytoscape software [13] will be used to analyze the PPIs to screen the hub genes.

## Materials and methods

### Microarray data

In this study, the data was obtained through the National Center for Biotechnology Information (NCBI: https://www.ncbi.nlm.nih.gov/geo/query/acc.cgi?acc=GSE33532) database. This dataset was based on the Affymetrix microarray GPL570 platform, which was submitted by Meister, et al. There are 100 samples in dataset GSE33532 including 80 NSCLC tissue samples and 20 normal tissue samples. Considering that 24 tumor samples do not have clear histopathological information, we selected 20 normal samples and 56 tumor samples for our analysis, including 40 AC samples and 16 SCC samples. These 56 samples were also classified as 32 samples in the first stage and 24 samples in the second stage according to the cancer infection (Table 1).

**Table 1:** Data description of cancer samples.

	The first stage	The second stage	Total
AC	24	16	40
SCC	8	8	16
Total	32	24	56

### Data pre-processing and clustering analysis of samples

For differential expression and correlation analysis, gene expression is seldom considered at the original counting level. Rather, it is common to convert the original data into a scale that is suitable for the library size. Here raw counts were transformed onto reads per kilobase of transcript per million (RPKM) values firstly. In the process of sample preparation and sequencing, there is no biological significance such as sample batches which will affect the expression of a single sample. Therefore, we need to standardize the data of each sample to ensure the similarity of data distribution. Here, normalization by the method of trimmed mean of M-value (TMM) was applied [14].

In the previous papers using GSE33532 datasets [15–18], the authors combined two different subtypes or stages of NSCLC into one single type directly and compared this type with normal samples. However, sample classification is an essential step in bioinformatic analysis. It is important to see whether genes are expressed at different level between different classifications. Therefore, in this study, we focused more on the information of sample classification. we used the plotMDS function in limma package to draw a multi-dimensional scaling (MDS) plot which showed the similarities and differences between different samples in an unsupervised way. And then we did the comparison based on the classification results. In our dataset, cancer subtypes and stages are two possible classification criterions and were therefore tested.

### Differential expression analysis

We followed the workflow in Bioconductor to find DEGs [19]. Firstly, we built a design matrix for pairwise comparisons based on classification information by makeContrasts function. Secondly, based on the limma linear fitting, the empirical Bayes moderation was carried out to infer the results of linear models [20]. P value < 0.05 was set by default to screen DEGs. The number of up- and down- regulated DEGs can be summarized. However, the empirical Bayes moderation is only successful in testing whether the differential expression differentiate from zero, which cannot guarantee that the differences found are large enough to have biological significance. Here, in order to get more meaningful conclusions, we used treat method, a t-test related to the minimum fold change, to screen DEGs. And the differential expression obtained is greater than a given threshold [21]. This method can also improve the existing false discovery rate and identify more biologically significant DEGs. Finally, the DEGs in multiple comparisons were extracted as the most important genes.

### GO functional and KEGG pathway analysis

In this study, we used DAVID (https://david.ncifcrf.gov/), a comprehensive set of functional annotation tool, to analyze GO function and KEGG pathway analysis of DEGs. It uses statistical methods to select the most prominent annotations in a large number of biological annotations, and the related information of their involvement in biological processes (BP), molecular function (MF), cell component (CC) and signal pathway can be found where *p* < 0.05 was considered to indicate a statistically significant difference.

### Integration of PPI network and identification of hub genes

We used STRING (https://string-db.org/), a database that collects and integrates known protein-protein interactions, to explore protein-protein interactions and construct PPI network. Through the plug-in network analysis in Cytoscape, the degree between nodes was calculated and the genes with the largest degree were selected to represent the hub genes which play important roles in the whole PPI network.

## Results

### Clustering analysis

**Fig 1:**
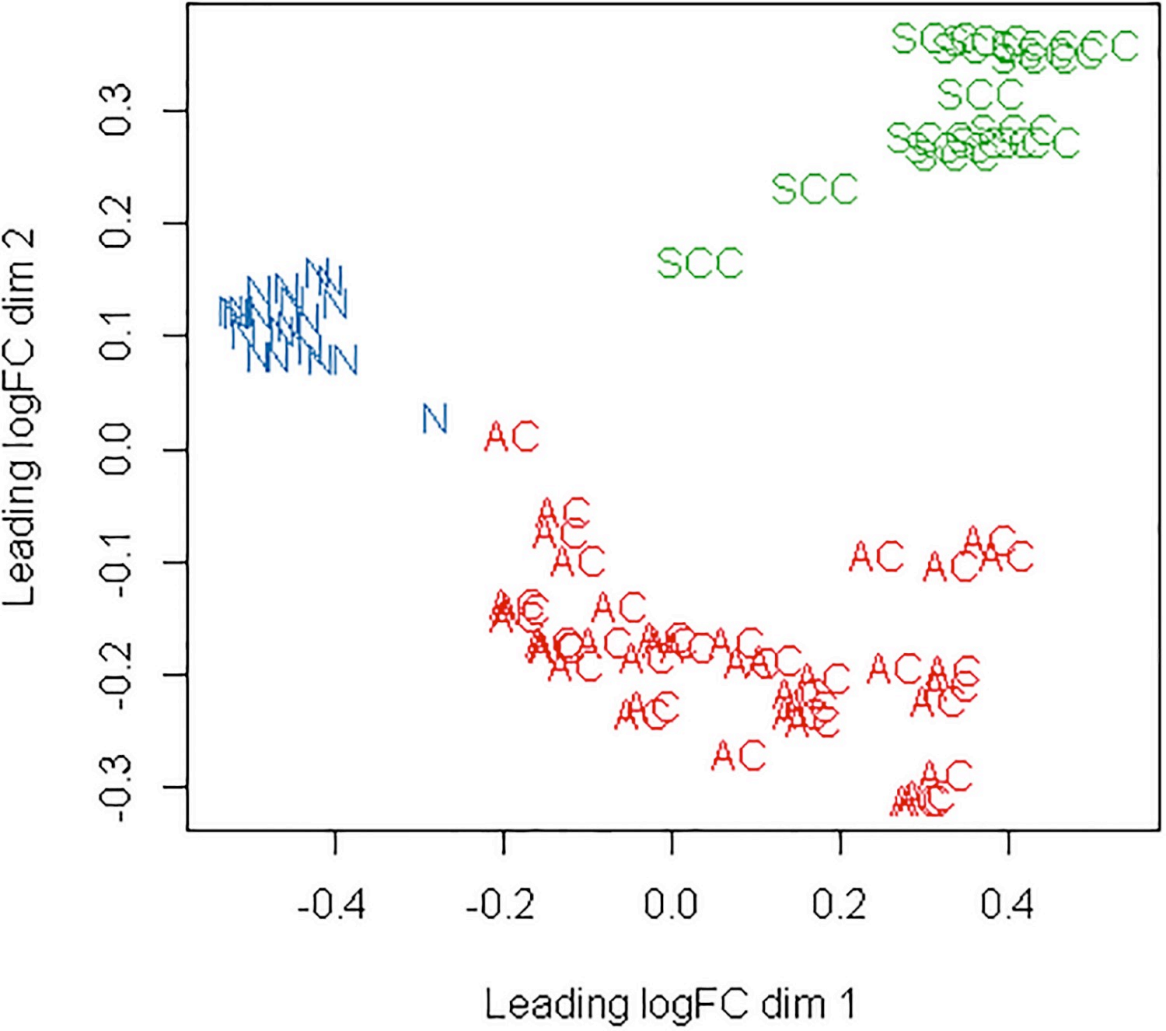
Clustering of samples.

Based on the two different classification criterions of the samples we found that, samples were clustered well within cancer subtypes over dimension 1 and 2, but the classification using the grouping defined by cancer stage was not good. The clustering result based on cancer subtypes was shown in Fig 1. The first dimension of the MDSplot explained the proportion of maximum changes in data. It showed that the transcription differences between AC versus N and SCC versus N were the greatest in the first dimension, which inspired us to compare the two cancer subtypes with the normal samples respectively to get more DEGs. Data sets of samples with poor clustering results may show little or no evidence of differential expression in downstream analysis. Therefore, we ignored the classification based on cancer stages.

### Differential expressed genes

Based on the empirical Bayes moderation, 13,629 DEGs were found including upregulated and downregulated for AC versus N, 14,271 DEGs were found for SCC versus N and 8,095 DEGs were found for AC versus SCC (Table 2, left).

**Table 2:** Number of up- and down-regulated genes for empirical Bayes and treat method.

	Empirical Bayes			Treat method		
	AC vs SCC	AC vs N	SCC vs N	AC vs SCC	AC vs N	SCC vs N
Down	3910	6588	7145	141	476	678
Not	14095	8561	7919	22012	21549	21105
Up	4185	7041	7126	37	165	407

In order to obtain more biologically significant conclusions, DEGs were screened according to treat method. The number of DEGs reduced to a total of 641 DEGs for AC versus N, 1,085 DEGs for SCC versus N and 178 DEGs for AC versus SCC when testing requires genes to have a Fold Change that is significantly greater than 1.2 (Table 2, right). Comparisons between AC versus N and SCC versus N resulted in a larger number of DEGs, which verified our conjecture from the MDS plot (Fig 1).

**Fig 2:**
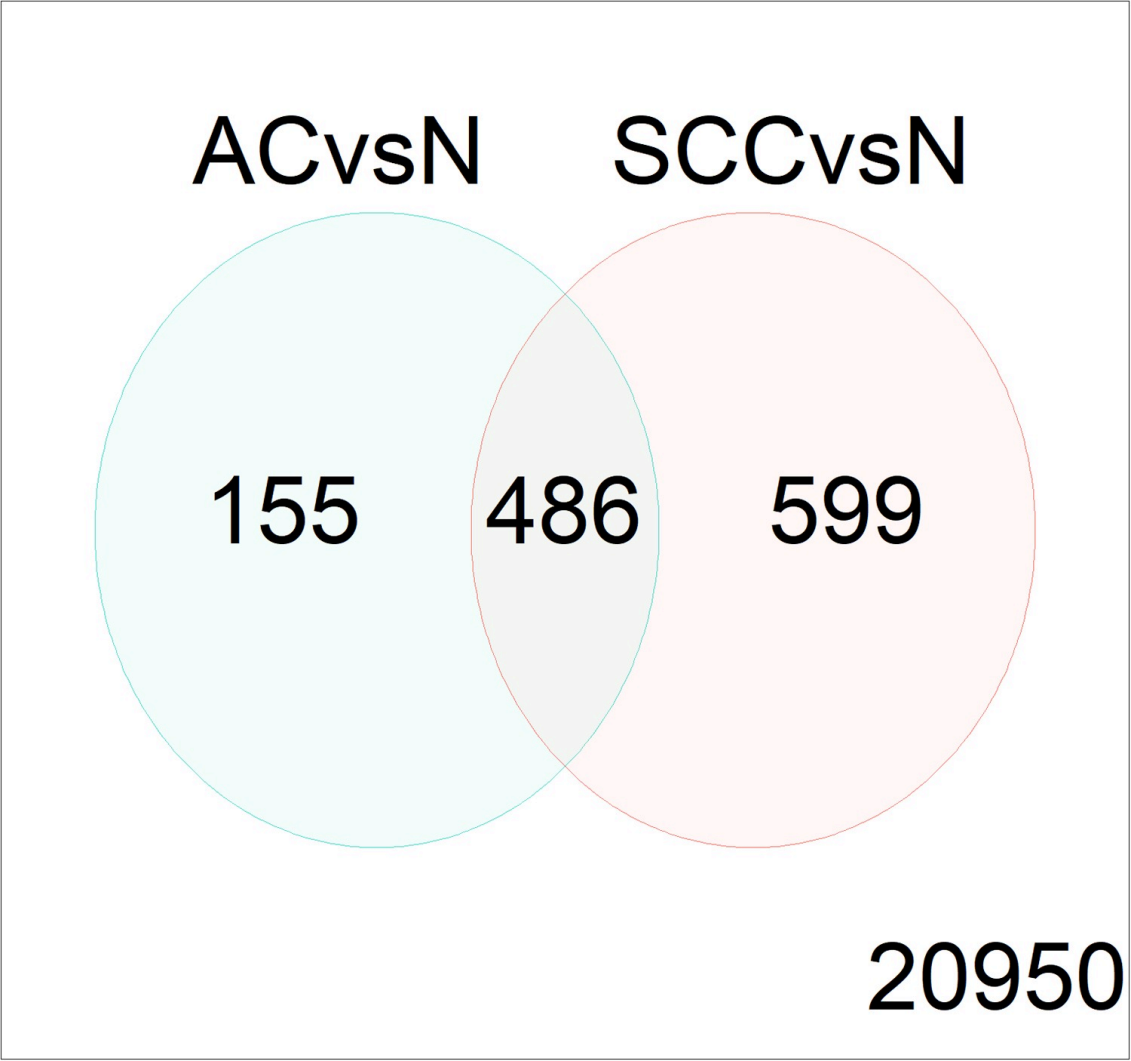
The number of DEGs in the comparison for the treat method.

Through integration of the DEGs in different contrasts, 486 DEGs including 116 upregulated and 370 downregulated DEGs in both AC versus N and SCC versus N were extracted by treat method (Fig 2), which were taken as the most significant genes associated with NSCLC.

### GO functional and KEGG pathway analysis

DAVID performed BP, MF and CC function analysis (Table 3) and KEGG pathway analysis (Table 4) on 116 upregulated DEGs and 370 downregulated DEGs, respectively.

**Table 3:** GO analysis of DEGs associated with NSCLC.

A.Upregulated			
Category	Term/gene function	P-value	Count
BP	GO:0007018 microtubule-based movement	5.53 × 10^−9^	9
BP	GO:0032467 positive regulation of cytokinesis	1.10 × 10^−5^	5
BP	GO:0007059 chromosome segregation	1.84 × 10^−4^	5
BP	GO:0000281 mitotic cytokinesis	3.22 × 10^−4^	4
BP	GO:0090307 mitotic spindle assembly	5.47 × 10^−4^	4
BP	GO:0007267 cell-cell signaling	8.26 × 10^−3^	4
CC	GO:0005737 cytoplasm	9.72 × 10^−3^	27
CC	GO:0005634 nucleus	7.08 × 10^−3^	26
CC	GO:0005654 nucleoplasm	2.73 × 10^−3^	18
CC	GO:0016020 membrane	4.34 × 10^−2^	11
CC	GO:0030496 midbody	4.41 × 10^−8^	9
CC	GO:0005871 kinesin complex	1.90 × 10^−8^	8
MF	GO:0005524 ATP binding	4.05 × 10^−6^	21
MF	GO:0003777 microtubule motor activity	9.37 × 10^−7^	7
MF	GO:0008574 ATP-dependent microtubule motor activity, plus-end-directed	9.68 × 10^−7^	5
MF	GO:0004222 metalloendopeptidase activity	2.71 × 10^−3^	5
MF	GO:0061630 ubiquitin protein ligase activity	9.04 × 10^−3^	5
MF	GO:0016887 ATPase activity	1.74 × 10^−2^	4
B.Downregulated			
Category	Term/gene function	P-value	Count
BP	GO:0043547 positive regulation of GTPase activity	4.74 × 10^−5^	24
BP	GO:0001525 angiogenesis	3.53 × 10^−6^	16
BP	GO:0007155 cell adhesion	7.85 × 10^−3^	16
BP	GO:0008285 negative regulation of cell proliferation	5.18 × 10^−3^	15
BP	GO:0035556 intracellular signal transduction	6.03 × 10^−3^	15
BP	GO:0006508 proteolysis	3.31 × 10^−2^	15
CC	GO:0016021 integral component of membrane	2.43 × 10^−3^	110
CC	GO:0005886 plasma membrane	1.19 × 10^−6^	106
CC	GO:0070062 extracellular exosome	4.76 × 10^−2^	59
CC	GO:0005887 integral component of plasma membrane	3.25 × 10^−7^	51
CC	GO:0005576 extracellular region	2.17 × 10^−4^	47
CC	GO:0005615 extracellular space	3.39 × 10^−3^	37
MF	GO:0005509 calcium ion binding	5.39 × 10^−4^	25
MF	GO:0005088 Ras guanyl-nucleotide exchange factor activity	9.62 × 10^−5^	10
MF	GO:0008201 heparin binding	1.11 × 10^−3^	10
MF	GO:0005215 transporter activity	1.61 × 10^−2^	9
MF	GO:0016887 ATPase activity	2.79 × 10^−2^	8
MF	GO:0004871 signal transducer activity	4.57 × 10^−2^	8

**Table 4:** KEGG analysis of DEGs associated with NSCLC.

A.Upregulated				
Pathway ID	Name	P-value	Count	Genes
hsa04110	Cell cycle	4.44 × 10^−6^	8	CCNB1, CDC6, CCNB2, MAD2L1, BUB1, TTK, BUB1B, CDC20
hsa04114	Oocyte meiosis	2.24 × 10^−2^	4	CCNB2, MAD2L1, BUB1, CDC20
hsa04914	Progesterone-mediated oocyte maturation	1.20 × 10^−2^	4	CCNB1, CCNB2, MAD2L1, BUB1
hsa03460	Fanconi anemia pathway	3.19 × 10^−2^	3	FANCI, BRIP1, UBE2T
B.Downregulated				
Pathway ID	Name	P-value	Count	Genes
hsa04261	Adrenergic signaling in cardiomyocytes	5.87 × 10^−6^	12	AGTR1, ADRB2, ADRB1, PLCB4, TNNC1, KCNE1, ADRA1A, SCN4B, RAPGEF4, SCN7A, ATP1A2, CACNA2D2
hsa04080	Neuroactive ligand-Receptor interaction	2.73 × 10^−3^	12	EDNRB, AGTR1, ADRB2, S1PR1, ADRB1, RXFP1, SSTR1, GRIA1, ADRA1A, CALCRL, NPY1R, VIPR1
hsa04024	cAMP signaling pathway	2.30 × 10^−2^	10	FXYD1, ADRB2, ADRB1, SSTR1, GRIA1, NPR1, RAPGEF4, HHIP, ATP1A2, NPY1R
hsa04022	cGMP-PKG signaling pathway	2.43 × 10^−3^	9	EDNRB, AGTR1, ADRB2, ADRB1, PLCB4, PDE5A, ADRA1A, NPR1, ATP1A2
hsa04270	Vascular smooth muscle contraction	1.77 × 10^−3^	8	RAMP3, RAMP2, AGTR1, PLCB4, PLA2G1B, ADRA1A, NPR1, CALCRL

As a result, it was shown that upregulated DEGs belonged to the component of cytoplasm, nucleus, nucleoplasm and other organelles, they had the molecular functions such as ATP binding, microtubule motor activity, ATP-dependent microtubule motor activity, plus-end-directed, participating in microtubule-based movement, positive regulation of cytokinesis, chromosome segregation and other biological processes (Table 3A). They were mainly involved in cell cycle, oocyte meiosis, progesterone-mediated oocyte maturation, Fanconi anemia pathway and other signaling pathways (Table 4A). While, downregulated DEGs belonged to the component of integral membrane, plasma membrane, extracellular exosome and other organelles, they had the molecular functions such as calcium ion binding, Ras guanyl-nucleotide exchange factor activity, heparin binding, participating in positive regulation of GTPase activity, angiogenesis, cell adhesion and other biological processes (Table 3B). They were mainly involved in adrenergic signaling in cardiomyocytes, neuroactive ligand-Receptor interaction, cGMP-PKG signaling pathway, vascular smooth muscle contraction and other signaling pathways (Table 4B).

### Integration of PPI network and identification of hub genes

**Fig 3:**
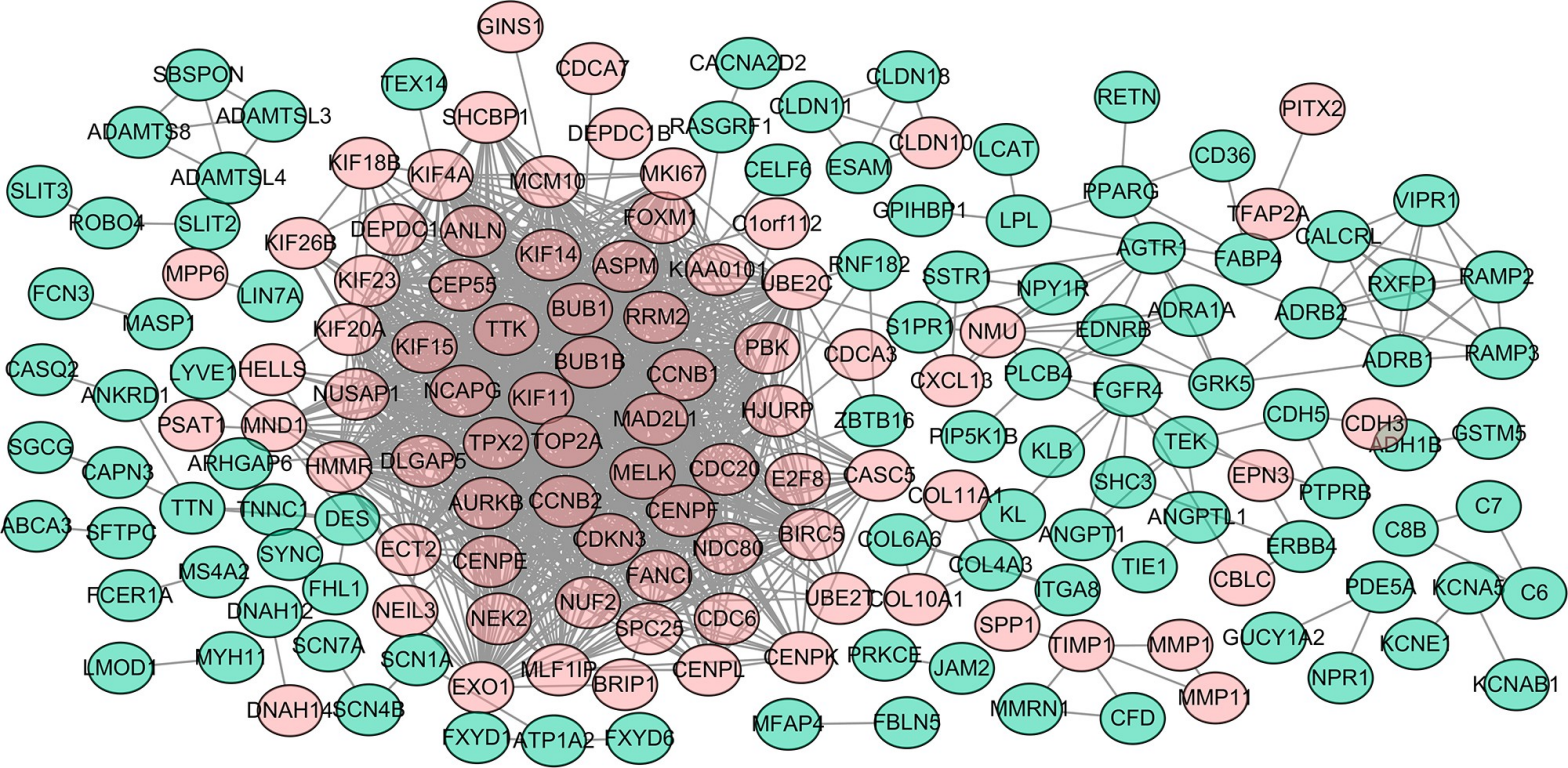
The PPI network.

After introducing all DEGs into STRING database, we constructed a PPI network which incorporated 436 nodes and 1,193 edges. We performed the subset of PPI network (Fig 3) for the DEGs with a combined score > 0.7 to determine the hub genes. As shown in Fig 3, blue nodes represented downregulated DEGs and red nodes represented upregulated DEGs. According to the degree of each gene, the top thirteen hub genes with the highest degrees were selected (Table 5), including BUBI mitotic checkpoint serine/threonine kinase (BUB1), Cyclin B1 (CCNB1), Mitotic arrest deficient 2 like 1 (MAD2L1), DNA topoisomerase 2-alpha (TOP2A), Kinesin family member 11 (KIF11), Cell division cycle 20 (CDC20), BUBI mitotic checkpoint serine/threonine kinase B (BUB1B), PDZ binding kinase(PBK), Abnormal spindle microtubule assembly(ASPM), Non-SMC condensin I complex subunit G(NCAPG), Centromere protein F(CENPF), TTK protein kinase(TTK) and Aurora kinase B(AURKB). Table 5 also showed that all the top thirteen hub genes were upregulated DEGs.

**Table 5:** Hub genes and rank of degrees.

Gene symbol	Description	Feature	Degree
BUB1	BUBI mitotic checkpoint serine/threonine kinase	up	52
CCNB1	Cyclin B1	up	51
MAD2L1	Mitotic arrest deficient 2 like 1	up	50
TOP2A	DNA topoisomerase 2-alpha	up	49
KIF11	Kinesin family member 11	up	49
CDC20	Cell divison cycle 20	up	49
BUB1B	BUBI mitotic checkpoint serine/threonine kinase B	up	49
PBK	PDZ binding kinase	up	48
ASPM	Abnormal spindle microtubule assembly	up	48
NCAPG	Non-SMC condensin I complex subunit G	up	48
CENPF	Centromere protein F	up	48
TTK	TTK protein kinase	up	48
AURKB	Aurora kinase B	up	48

## Discussion

NSCLC has been a serious threat to the public health worldwide. It is important to identify genes which express differentially between subtypes and normal cases, predict their underlying functions, pathways and construct PPI network for the diagnosis and treatment of NSCLC.

In the present study, based on the expression profiles of GSE33532, which is associated with the early stage of NSCLC, selection of DEGs and bioinformatics analysis were performed. In our process of data analysis, we focused more on the classification of samples which was an essential step of bioinformatics analysis and found that samples were clustered well within cancer subtypes. Considering the difference between the two subtypes of NSCLC, we compared both cancer subtypes with normal samples, respectively. And then we took intersection when selecting DEGs for subsequent analysis. We eventually found 116 upregulated DEGs and 370 downregulated DEGs. To obtain further analysis of these DEGs, we performed GO functional analysis and KEGG pathway analysis.

We found that the upregulated DEGs mainly participated in four pathways, the top three are cell cycle, oocyte meiosis nad progesterone-mediated oocyte maturation, which were consistent with previous results using the same dataset. Besides, we also found a new pathway through data analysis, the Fanconi anemia complex I that functions to activate FANCD2 and FANCI by mono-ubiquitinating the protein following response to DNA damage. The Fanconi anemia pathway is a major mechanism of homologous recombination DNA repair. DNA-repair deficiencies have been considered of interest in lung cancer prevention, given the persistence of damage produced by cigarette smoke in this setting, as well as in treatment, given potential increased efficacy of DNA-damaging drugs [22–24].

The downregulated DEGs mainly participated in five pathways, including adrenergic signaling in cardiomyocytes, neuroactive ligand-Receptor interaction, cAMP signaling pathway, cGMP-PKG signaling pathway and vascular smooth muscle contraction. All these pathways were not mentioned in the previous studies but played important roles in lung cancer or other disease. Beta-adrenergic signaling has been found to regulate multiple cellular processes that contribute to the initiation and progression of cancer, including inflammation, angiogenesis, apoptosis/anoikis, cell motility and trafficking, activation of tumor-associated viruses, DNA damage repair, cellular immune response and epithelial–mesenchymal transition [25]. The increase in cAMP levels activates target molecules, such as cAMP-dependent protein kinase (protein kinase A, PKA), exchange protein directly activated by cAMP (Epac) and cyclic nucleotide-gated ion channels [26]. These target effector molecules regulate various cellular responses, including metabolism, gene expression, proliferation and apoptosis. Various alterations to key molecules of the cAMP signaling pathway have been observed in lung cancer, and phosphodiesterase inhibitors have been shown to synergize with cisplatin to induce apoptosis in a broad panel of human lung cancer cell lines [27]. Down-regulation of cGMP/PKG-mediated signaling pathways often occurs during tumorigenesis and cell transformation, the activation of the cGMP-dependent enzyme protein kinase G (PKG) can play an important role in inhibiting cell proliferation and inducing apoptosis [28]. Vascular smooth muscle (VSM) is a major component of the tunica media of blood vessels, and an important regulator of vascular function. VSM contraction plays an important role in the regulation of peripheral vascular resistance and blood pressure, and vascular dysfunction, excessive vasoconstriction, and vasospasm could lead to major cardiovascular disorders such as hypertension and coronary artery disease [29].

Through the PPI network, we selected thirteen hub genes. Most of these hub genes were reported by previous studies to participate in the corresponding functions during the infection of NSCLC [30–43]. We also found five new hub genes that were not reported in previous references using dataset GSE33532, including TOP2A, PBK, ASPM, NCAPG and TTK. Four of them had great impacts on lung cancer based on experimental results, which was summarized as follows. The overexpression of TOP2A in NSCLC tissues is related to lymph node metastasis, which can promote cell proliferation and invasion [33]. PBK, also known as TOPK, is a potential therapeutic target in lung cancer that promotes cell migration by modulating a PI3K/PTEN/AKT-dependent signaling pathway. High PBK expression, either alone or in combination with a low level of PTEN, may serve as a prognostic marker for lung cancer [37]. Suberoylanilide hydroxamic acid significantly enhanced the tumor initiating capacity and the expression of malignant genes such as ASPM in the remaining living ALDH cells, which can suppress the growth of tumor xenografts and decreases the lung cancer stem cell population in vivo [38]. The non-SMC condensin I complex subunit G (NCAPG) that organizes the coiling topology of individual chromatids, represents an overexpressed antigen in various types of cancer, and also contributes to restructuring chromatin into rod-shaped mitotic chromosomes and ensuring the segregation of sister chromatid during cell division [39]. The expression of TTK in lung cancer tissues is significantly different from that in smokers and non-smokers, which is consistent with the important role of TTK in smoking-induced lung cancer. TTK is a candidate target gene for chemical prevention and treatment of lung cancer in smokers [42].

Further, six of the selected hub genes including BUB1, CCNB1, MAD2L1, CDC20, BUB1B and TTK were found to participated in the same cell cycle pathway. It was also shown by the previous study that these hub genes served as a regulatory protein at multiple checkpoints in the cell cycle pathway. Cell cycle pathway is the key pathway of lung cancer and regulatory proteins located in cell cycle signaling pathway play an important role in the mechanism of lung cancer [44–46].

In conclusion, the present study provides a broader analysis of DEGs for NSCLC which contributes to exploration NSCLC pathogenesis and may serve as potential biomarkers for future research on early NSCLC detection. However, current research is theoretical analysis based on data, prospective clinical studies remains to be an important next step of investigation.

## Supporting information

S1 Table The 486 included differentially expressed genes and their related information. (DOCX)

## References

[1] Siegel RL, Miller KD and Jemal A. Cancer statistics, 2018. Ca A Cancer Journal for Clinicians. 2018;60(5):277–300. 10.3322/caac.20073 20610543

[2] Raponi M. Gene expression signatures for predicting prognosis of squamous cell and adenocarcinomas of the lung. Cancer Research.2006;66(15):7466–7472. 10.1158/0008-5472.CAN-06-1191 16885343

[3] Spira A and Ettinger DS. Multidisciplinary management of lung cancer. New England Journal of Medicine. 2004;350:2008–2010. 10.1056/NEJM200405063501921 14736930

[4] Dalmay T and Edwards DR. MicroRNAs and the hallmarks of cancer. Oncogene. 2006;25(46):6170–6175. 10.1038/sj.onc.1209911 17028596

[5] Liang B, Li C and Zhao J. Identification of key pathways and genes in colorectal cancer using bioinformatics analysis. Medical Oncology. 2016;33(10):111 10.1007/s12032-016-0829-6 27581154

[6] Kulasingam V and Diamandis EP. Strategies for discovering novel cancer biomarkers through utilization of emerging technologies. Nature Clinical Practice Oncology. 2008;5:588–599. 10.1038/ncponc1187 18695711

[7] Robinson MD, Mccarthy DJ and Smyth GK. EdgeR: a Bioconductor package for differential expression analysis of digital gene expression data. Bioinformatics. 2010;26(1):139–140. 10.1093/bioinformatics/btp616 19910308 PMC2796818

[8] Ritchie ME, Phipson B, Wu D et al Limma powers differential expression analyses for RNA-sequencing and microarray studies. Nucleic Acids Research. 2015;43(7):e47 10.1093/nar/gkv007 25605792 PMC4402510

[9] Huang WD, Sherman BT and Lempicki RA. Systematic and integrative analysis of large gene lists using DAVID bioinformatics resources. Nat Protoc. 2009;4(1):44–57. 10.1038/nprot.2008.211 19131956

[10] Martucci D, Masseroli M and Pinciroli F. Gene ontology application to genomic functional annotation, statistical analysis and knowledge mining. Studies in health technology and informatics. 2004;102:108–131. 15853267

[11] Ogata H, Goto S, Sato K et al KEGG: Kyoto Encyclopedia of Genes and Genomes. Nucleic Acids Research. 2000;28(1):27–30. 10.1093/nar/28.1.27 10592173 PMC102409

[12] Szklarczyk D, Morris JH, Cook H et al The STRING database in 2017: quality-controlled protein-protein association networks made broadly accessible. Nucleic Acids Research.2017;45(D1):D362–D368. 10.1093/nar/gkw937 27924014 PMC5210637

[13] Saito R, Smoot ME, Ono K et al A travel guide to Cytoscape plugins. Nature Methods. 2012;9(11):1069–1076. 10.1038/nmeth.2212 23132118 PMC3649846

[14] Robinson MD and Oshlack A. A scaling normalization method for differential expression analysis of RNA-seq data. Genome Biology. 2010;11(3):1–9. 10.1186/gb-2010-11-3-r25 PMC2864565 20196867

[15] Huang H, Huang QD, Tang TY et al Differentially expressed gene screening, biological function enrichment, and correlation with prognosis in non-small cell lung cancer. Medicine Science Monitor. 2019;25:4333–4341. 10.12659/MSM.916962 PMC6582684 31181055

[16] Li Y, Gu J, Xu FK et al Transcriptomic and functional network features of lung squamous cell carcinoma through integrative analysis of GEO and TCGA data. Scientific Reports. 2018;8(1):15834 10.1038/s41598-018-34160-w 30367091 PMC6203807

[17] Tang Q, Zhang HM, Kong M et al Hub genes and key pathways of non-small lung cancer identified using bioinformatics. Oncology Letters. 2018;16(2):2344–2354. 10.3892/ol.2018.8882 30008938 PMC6036325

[18] Li SC, Xuan YP, Gao B et al Identification of an eight-gene prognostic signature for lung adenocarcinoma. Cancer Management and Research. 2018;10:3383–3392. 10.2147/CMAR.S173941 30237740 PMC6138967

[19] Law CW, Alhamdoosh M, Su S et al RNA-seq analysis is easy as 1-2-3 with limma, Glimma and edgeR. F1000research. 2016;5:1408 10.12688/f1000research.9005.1 PMC4937821 27441086

[20] Smyth GK. Linear models and empirical Bayes methods for assessing differential expression in microarray experiments. Stat Appl Genet Mol Biol. 2004;3(3):Article ID 3. 10.2202/1544-6115.1027 16646809

[21] Mccarthy DJ and Smyth GK. Testing significance relative to a fold-change threshold is a TREAT. Bioinformatics. 2009;25(6):765–771. 10.1093/bioinformatics/btp053 19176553 PMC2654802

[22] Smogorzewska A, Matsuoka S, Vinciguerra P et al Identification of the FANCI protein, a monoubiquitinated FANCD2 paralog required for DNA repair. Cell. 2007;129(2):289–301. 10.1016/j.cell.2007.03.009 17412408 PMC2175179

[23] Taniguchi T, D’Andrea AD. Molecular pathogenesis of Fanconi anemia:recent progress. Blood. 2006;107(11):4223–4233. 10.1182/blood-2005-10-4240 16493006

[24] Duan W, Gao L, Aguila B et al Fanconi Anemia Repair Pathway Dysfunction, a Potential Therapeutic Target in Lung Cancer. Front Oncol. 2014;4:368 10.3389/fonc.2014.00368 25566506 PMC4271581

[25] Cole SW, Sood AK. Molecular pathways: beta-adrenergic signaling in cancer. Clinical Cancer Research An Official Journal of the American Association for Cancer Research. 2012;18(5):1201 10.1158/1078-0432.CCR-11-0641 22186256 PMC3294063

[26] Fimia GM, Sassone-Corsi P. Cyclic AMP signalling. Journal of Cell Science. 2001;114:1971–1972. 11493633 10.1242/jcs.114.11.1971

[27] Park JY, Juhnn YS. cAMP signaling increases histone deacetylase 8 expression via the Epac2-Rap1A-Akt pathway in H1299 lung cancer cells. Experimental and Molecular Medicine. 2017;49(2):e297 10.1038/emm.2016.152 28232663 PMC5336561

[28] Deguchi A, Das KK, Xing SW et al Down-regulation of the cGMP/PKG pathway in primary human colon cancers and cancer cell lines. Cancer Research. 2005;65(9):2330.15781647

[29] Liu Z, Khalil RA. Evolving Mechanisms of Vascular Smooth Muscle Contraction Highlight Key Targets in Vascular Disease. Cancer Research. 2018;(153):91–122. 10.1016/j.bcp.2018.02.012 PMC5959760 29452094

[30] Shigeishi H, Oue N, Kuniyasu H et al Expression of Bub1 gene correlates with tumor proliferating activity in human gastric carcinomas. Pathobiology. 2001;69(1):24–29. 10.1159/000048754 11641614

[31] Soria J, Jang SJ, Khuri FR et al Advances in brief overexpression of Cyclin B1 in early-stage non-small cell lung cancer and its clinical implication 1. Cancer Research. 2000;60(15):4000–4004. 10945597

[32] Guo Y, Zhang X, Yang M et al Functional evaluation of missense variations in the human MAD1L1 and MAD2L1 genes and their impact on susceptibility to lung cancer. Journal of Medical Genetics. 2010;47(9):616–622. 10.1136/jmg.2009.074252 20516147

[33] Huang H, Liu J, Meng Q et al Multidrug resistance protein and topoisomerase 2 alpha expression in non-small cell lung cancer are related with brain metastasis postoperatively. International Journal of Clinical and Experimental Pathology. 2015;8(9):11537–11542. 26617887 PMC4637703

[34] Schneider MA, Christopoulos P, Muley T et al AURKA, DLGAP5, TPX2, KIF11 and CKAP5: Five specific mitosis-associated genes correlate with poor prognosis for non-small cell lung cancer patients. International Journal of Oncology. 2017;50(2):365–372. 10.3892/ijo.2017.3834 28101582 PMC5238780

[35] Kato T, Daigo Y, Aragaki M et al Overexpression of CDC20 predicts poor prognosis in primary non-small cell lung cancer patients. Journal of Surgical Oncology. 2012;106(4):423–430. 10.1002/jso.23109 22488197

[36] Chen H, Lee J, Kljavin NM et al Abstract 2259: Requirement for BUB1B in tumor progression of lung adenocarcinoma. Cancer Research. 2015;75(S15):2259. 10.18632/genesandcancer.53 PMC4426948 26000094

[37] Shih MC, Chen JY, Wu YC et al TOPK/PBK promotes cell migration via modulation of the PI3K/PTEN/AKT pathway and is associated with poor prognosis in lung cancer. Oncogene. 2012;31(19):2389–2400. 10.1038/onc.2011.419 21996732

[38] Kuo WY, Wu CY, Hwu L et al Enhancement of tumor initiation and expression of KCNMA1, MORF4L2 and ASPM genes in the adenocarcinoma of lung xenograft after vorinostat treatment. Oncotarget. 2015;6(11):8663–8675. 10.18632/oncotarget.3536 25796627 PMC4496174

[39] Liu W, Liang B, Liu H et al Overexpression of non-SMC condensin I complex subunit G serves as a promising prognostic marker and therapeutic target for hepatocellular carcinoma. International Journal of Molecular Medicine. 2017;40(3):731–738. 10.3892/ijmm.2017.3079 28737823 PMC5547945

[40] Varis A, Salmela AL, Kallio MJ. CENPF (mitosin) is more than a mitotic marker. Chromosoma (Berlin). 2006;115(4):288–295. 10.1007/s00412-005-0046-0 16565862

[41] Liao, H et al CENPF is a protein of the nuclear matrix that assembles onto kinetochores at late G2 and is rapidly degraded after mitosis. The Journal of Cell Biology. 1995;130(3):507–518. 10.1083/jcb.130.3.507 7542657 PMC2120529

[42] Teresa LM, Tatiana D, Melissa R et al Gene Expression Signature of Cigarette Smoking and Its Role in Lung Adenocarcinoma Development and Survival. PLoS ONE. 2008;3(2):e1651 10.1371/journal.pone.0001651 18297132 PMC2249927

[43] Smith SL, Bowers NL, Betticher DC et al Overexpression of aurora B kinase (AURKB) in primary non-small cell lung carcinoma is frequent, generally driven from one allele, and correlates with the level of genetic instability. British journal of cancer. 2005;93(6):719–729. 10.1038/sj.bjc.6602779 16222316 PMC2361619

[44] Williams GH and Stoeber K. The cell cycle and cancer. Proceedings of the National Academy of Sciences of the United States of America. 2012;226(2):352–364. 10.1002/path.3022 21990031

[45] Wang W, Spitz MR, Yang H et al Genetic variants in cell cycle control pathway confer susceptibility to lung cancer. Clinical Cancer Research: An Official Journal of the American Association for Cancer Research. 2007;13(19):5974–5981. 10.1158/1078-0432.CCR-07-0113 17908995

[46] Caputi M, Russo G, Esposito V et al Role of cell-cycle regulators in lung cancer. Journal of Cellular Physiology. 2005;205(3):319–327. 10.1002/jcp.20424 15965963

