# Supplementary material for: Identifying candidate diagnostic markers for early stage of non-small cell lung cancer
Source: PLoS One. 2019 Nov 14;14(11):e0225080. doi: 10.1371/journal.pone.0225080 (PMC6855900; doi:10.1371/journal.pone.0225080)
Supplement: S1 Table — (DOCX) [file pone.0225080.s001.docx]

Table S1: The 486 included differentially expressed genes and their related information

| Gene ID | Gene Symbol | Log-FC  (AC .vs. N) | Log-FC  (SCC .vs. N) | GO term | Pathway ID |
| --- | --- | --- | --- | --- | --- |
| 205326_at | RAMP3 | -0.47 | -0.56 | GO:0016021;  GO:0005886;  GO:0005887; | hsa04270 |
| 205779_at | RAMP2 | -0.48 | -0.42 | GO:0001525;  GO:0016021;  GO:0005886;  GO:0005887; | hsa04270 |
| 206311_s_at | PLA2G1B | -0.68 | -0.92 | GO0007125;  GO:0005576;  GO:0005615; GO:0035556； GO:0005509； | hsa04270 |
| 203296_s_at | ATP1A2 | -0.71 | -0.88 | GO:0016021;  GO:0005886;  GO:0016887 | hsa04261; hsa04024;  hsa04022 |
| 205651_x_at | RAPGEF4 | -0.50 | -0.64 | GO:0043547;  GO:0005886;  GO:0000588 | hsa04261;  hsa04024 |
| 206170_at | ADRB2 | -0.61 | -0.52 | GO:0016021;  GO:0005886;  GO:0005887 | hsa04261;  hsa04080;  hsa04024;  hsa04022 |
| 203895_at | PLCB4 | -0.64 | -0.59 | GO:0005509;  GO:0004871;  GO:0035556 | hsa04261;  hsa04022;  hsa04270 |
| 205357_s_at | AGTR1 | -0.69 | -0.86 | GO:0016021;  GO:0005886;  GO:0005887 | hsa04261;  hsa04080;  hsa04022;  hsa04270 |
| 229309_at | ADRB1 | -0.99 | -0.93 | GO:0043547;  GO:0005886;  GO:0005887;  GO:0005088 | hsa04261;  hsa04024;  hsa04022;  hsa04080 |
| 237390_at | ADRA1A | -1.08 | -1.13 | GO0007125;  GO:0008285;  GO:0016021;  GO:0005886;  GO:0005887;  GO:0035556 | hsa04261;  hsa04022;  hsa04270;  hsa04080 |
| 236407_at | KCNE1 | -0.55 | -0.49 | GO:0016021;  GO:0005886; | hsa04261 |
| 204811_s_at | CACNA2D2 | -0.57 | -0.84 | GO:0005886 | hsa04261 |
| 228504_at | SCN7A | -0.62 | -0.96 | GO:0005886 | hsa04261 |
| 236359_at | SCN4B | -0.70 | -0.66 | GO:0016021;  GO:0005886; | hsa04261 |
| 209904_at | TNNC1 | -0.83 | -1.01 | GO:0005509 | hsa04261 |
| 203362_s_at | MAD2L1 | 0.57 | 0.75 |  | hsa04110; hsa04914;  hsa04114 |
| 202705_at | CCNB2 | 0.52 | 0.72 | GO:0005634 | hsa04110; hsa04914;  hsa04114 |
| 202870_s_at | CDC20 | 0.61 | 0.85 | GO:0005737;  GO:0005654 | hsa04110;  hsa04114 |
| 214710_s_at | CCNB1 | 0.61 | 0.77 | GO:0005634 | hsa04110;  hsa04914 |
| 209642_at | BUB1 | 0.59 | 0.83 | GO:0005737;  GO:0005654;  GO:0016020;  GO:0005524 | hsa04110;  hsa04914;  hsa04114 |
| 204822_at | TTK | 0.59 | 0.87 | GO:0016020;  GO:0005524 | hsa04110 |
| 203755_at | BUB1B | 0.57 | 0.81 |  | hsa04110 |
| 203968_s_at | CDC6 | 0.49 | 0.76 | GO:0032467;  GO:0005654  GO:0005524 | hsa04110 |
| 206331_at | CALCRL | -0.43 | -0.42 | GO:0001525;  GO:0016021;  GO:0005886;  GO:0005887 | hsa04080;  hsa04270 |
| 204271_s_at | EDNRB | -0.47 | -0.62 | GO:0016021;  GO:0005886;  GO:0005887 | hsa04080;  hsa04022 |
| 205440_s_at | NPY1R | -0.54 | -0.77 | GO:0016021;  GO:0005886;  GO:0005887 | hsa04080;  hsa04024 |
| 204642_at | S1PR1 | -0.42 | -0.43 | GO:0043547;  GO:0001525;  GO:0007155;  GO:0016021;  GO:0005886 | hsa04080 |
| 205019_s_at | VIPR1 | -0.59 | -0.63 | GO:0016021;  GO:0005886;  GO:0005887 | hsa04080 |
| 231804_at | RXFP1 | -0.73 | -0.68 | GO:0016021;  GO:0005886 | hsa04080 |
| 32625_at | NPR1 | -0.42 | -0.44 | GO:0016021;  GO:0005886;  GO:0005887;  GO:0035556 | hsa04024;  hsa04022;  hsa04270 |
| 235591_at | SSTR1 | -0.82 | -0.99 | GO:0008285;  GO:0005886;  GO:0005887 | hsa04024;  hsa04080 |
| 209793_at | GRIA1 | -0.84 | -0.80 | GO0007125;  GO:0016021;  GO:0005886; | hsa04024;  hsa04080 |
| 205384_at | FXYD1 | -0.48 | -0.53 | GO:0016021;  GO:0005886;  GO:0005887 | hsa04024 |
| 230135_at | HHIP | -0.72 | -0.99 | GO:0005887;  GO:0005576; | hsa04024 |
| 227088_at | PDE5A | -0.43 | -0.51 | GO0007125 | hsa04022 |
| 235609_at | BRIP1 | 0.51 | 0.60 | GO:0005737;  GO:0005634;  GO:0005524 | hsa03460: |
| 213007_at | FANCI | 0.44 | 0.64 | GO:0005737;  GO:0005654;  GO:0016020 | hsa03460: |
| 223229_at | UBE2T | 0.62 | 0.73 | GO:0005737;  GO:0005634;  GO:0061630;  GO:0005524 | hsa03460 |
| 218469_at | GREM1 | 1.07 | 1.18 | GO:0007267 |  |
| 206504_at | CYP24A1 | 1.05 | 0.53 | GO:0005654 |  |
| 37892_at | COL11A1 | 1.04 | 1.13 |  |  |
| 204475_at | MMP1 | 0.90 | 1.26 | GO:0004222 |  |
| 222608_s_at | ANLN | 0.90 | 1.16 | GO:0000281;  GO:0005654 |  |
| 223278_at | GJB2 | 0.89 | 1.25 | GO:0007267 |  |
| 203764_at | DLGAP5 | 0.86 | 1.08 | GO:0005737;  GO:0005634 |  |
| 219148_at | PBK | 0.85 | 1.14 | GO:0005654;  GO:0005524 |  |
| 1552767_a_at | HS6ST2 | 0.85 | 0.64 | GO:0005654 |  |
| 204580_at | MMP12 | 0.84 | 1.21 | GO:0004222 |  |
| 205941_s_at | COL10A1 | 0.84 | 0.68 |  |  |
| 219936_s_at | GPR87 | 0.81 | 1.37 |  |  |
| 223062_s_at | PSAT1 | 0.79 | 0.89 | GO:0005737 |  |
| 204653_at | TFAP2A | 0.79 | 0.92 | GO:0005634 |  |
| 218542_at | CEP55 | 0.78 | 1.00 |  |  |
| 225655_at | UHRF1 | 0.76 | 0.82 | GO:0061630 |  |
| 223381_at | NUF2 | 0.75 | 0.97 | GO:0005634;  GO:0016020 |  |
| 206023_at | NMU | 0.75 | 0.93 |  |  |
| 219918_s_at | ASPM | 0.73 | 0.92 | GO:0005737;  GO:0005634;  GO:0030496 |  |
| 204641_at | NEK2 | 0.73 | 0.93 | GO:0007059;  GO:0090307;  GO:0005737;  GO:0005634;  GO:0030496;  GO:0005524 |  |
| 226980_at | DEPDC1B | 0.73 | 0.99 |  |  |
| 205328_at | CLDN10 | 0.72 | 0.61 | GO:0005737 |  |
| 201292_at | TOP2A | 0.72 | 0.92 | GO:0005634;  GO:0005654;  GO:0005524 |  |
| 219990_at | E2F8 | 0.71 | 0.67 | GO:0005634 |  |
| 203889_at | SCG5 | 0.71 | 0.52 |  |  |
| 207165_at | HMMR | 0.70 | 0.80 | GO:0016020 |  |
| 231771_at | GJB6 | 0.70 | 1.64 |  |  |
| 209875_s_at | SPP1 | 0.69 | 0.65 |  |  |
| 224428_s_at | CDCA7 | 0.67 | 0.60 |  |  |
| 209125_at | KRT6A | 0.66 | 1.74 |  |  |
| 206134_at | ADAMDEC1 | 0.66 | 0.86 | GO:0004222 |  |
| 238617_at | KIF26B | 0.66 | 0.53 | GO:0007018;  GO:0003777;  GO:0005524;  GO:0008574;  GO:0005871 |  |
| 206102_at | GINS1 | 0.65 | 0.81 |  |  |
| 207558_s_at | PITX2 | 0.65 | 1.15 | GO:0005737;  GO:0005634 |  |
| 201250_s_at | SLC2A1 | 0.65 | 1.00 | GO:0030496 |  |
| 204962_s_at | SLC35F6  ///CENPA | 0.65 | 0.85 |  |  |
| 204444_at | KIF11 | 0.64 | 0.78 | GO:0007018;  GO:0007059;  GO:0090307;  GO:0005737;  GO:0016020;  GO:0005871;  GO:0008574;  GO:0005524 |  |
| 218662_s_at | NCAPG | 0.63 | 0.79 | GO:0005737;  GO:0016020 |  |
| 218355_at | KIF4A | 0.62 | 0.88 | GO:0007018;  GO:0005871;  GO:0008574;  GO:0005524 |  |
| 202095_s_at | BIRC5 | 0.62 | 0.86 | GO:0007059;  GO:0090307;  GO:0005737;  GO:0005634;  GO:0030496 |  |
| 206224_at | CST1 | 0.61 | 0.81 |  |  |
| 209891_at | SPC25 | 0.61 | 0.96 | GO:0007059 |  |
| 242283_at | DNAH14 | 0.61 | 0.71 |  |  |
| 227452_at | LINC00673  ///LINC00511 | 0.61 | 0.81 |  |  |
| 204162_at | NDC80 | 0.61 | 0.79 | GO:0016020 |  |
| 210052_s_at | TPX2 | 0.61 | 0.82 | GO:0090307;  GO:0005654 |  |
| 223700_at | MND1 | 0.61 | 0.82 | GO:0005634 |  |
| 225681_at | CTHRC1 | 0.60 | 0.64 | GO:0005737 |  |
| 201890_at | RRM2 | 0.60 | 0.80 | GO:0005634 |  |
| 222958_s_at | DEPDC1 | 0.60 | 0.86 | GO:0005654 |  |
| 207828_s_at | CENPF | 0.59 | 0.73 |  |  |
| 204825_at | MELK | 0.59 | 0.76 | GO:0005737;  GO:0005634;  GO:0005524 |  |
| 220651_s_at | MCM10 | 0.59 | 0.80 | GO:0005737 |  |
| 205242_at | CXCL13 | 0.59 | 0.83 | GO:0007267 |  |
| 242881_x_at | DUXAP10 | 0.59 | 0.77 |  |  |
| 229802_at | WISP1 | 0.58 | 0.63 | GO:0007267;  GO:0005737 |  |
| 223895_s_at | EPN3 | 0.58 | 0.59 | GO:0005654 |  |
| 218755_at | KIF20A | 0.58 | 0.69 | GO:0007018;  GO:0000281;  GO:0030496;  GO:0005871;  GO:0003777;  GO:0005524;  GO:0016887 |  |
| 219502_at | NEIL3 | 0.57 | 0.58 | GO:0005654 |  |
| 209714_s_at | CDKN3 | 0.56 | 0.74 | GO:0005737;  GO:0005634 |  |
| 232202_at | FAM83B | 0.55 | 1.21 | GO:0005737;  GO:0016020 |  |
| 212022_s_at | MKI67 | 0.55 | 0.61 | GO:0016020 |  |
| 202503_s_at | KIAA0101 | 0.55 | 0.65 | GO:0005634 |  |
| 219306_at | KIF15 | 0.55 | 0.67 | GO:0007018;  GO:0016020;  GO:0005871;  GO:0003777;  GO:0005524;  GO:0016887 |  |
| 202954_at | UBE2C | 0.55 | 0.69 | GO:0005737;  GO:0005634;  GO:0061630;  GO:0005524 |  |
| 228323_at | KNL1 | 0.55 | 0.82 | GO:0005654 |  |
| 220431_at | TMPRSS11E | 0.53 | 0.87 |  |  |
| 218726_at | HJURP | 0.53 | 0.68 | GO:0007059;  GO:0005737;  GO:0005634 |  |
| 204709_s_at | KIF23 | 0.53 | 0.84 | GO:0007018;  GO:0032467;  GO:0000281;  GO:0005654;  GO:0030496;  GO:0005871;  GO:0003777;  GO:0005524;  GO:0016887 |  |
| 229276_at | IGSF9 | 0.53 | 0.72 |  |  |
| 206364_at | KIF14 | 0.52 | 0.64 | GO:0007018;  GO:0032467;  GO:0030496;  GO:0005871;  GO:0003777;  GO:0005524;  GO:0008574;  GO:0016887 |  |
| 205046_at | CENPE | 0.52 | 0.70 | GO:0007018;  GO:0003777;  GO:0005524 |  |
| 203256_at | CDH3 | 0.52 | 0.79 |  |  |
| 227801_at | TRIM59 | 0.52 | 0.58 | GO:0061630 |  |
| 222039_at | KIF18B | 0.51 | 0.68 | GO:0005871;  GO:0003777;  GO:0005524;  GO:0008574;  GO:0007018 |  |
| 218960_at | TMPRSS4 | 0.51 | 0.89 |  |  |
| 241994_at | XDH | 0.51 | 0.46 |  |  |
| 219493_at | SHCBP1 | 0.51 | 0.56 |  |  |
| 222848_at | CENPK | 0.51 | 0.58 | GO:0005634 |  |
| 209464_at | AURKB | 0.49 | 0.70 | GO:0032467;  GO:0005654;  GO:0030496;  GO:0005524 |  |
| 238439_at | ANKRD22 | 0.47 | 0.73 |  |  |
| 235476_at | TRIM59  ///IFT80 | 0.47 | 0.53 |  |  |
| 203878_s_at | MMP11 | 0.47 | 0.48 | GO:0004222 |  |
| 219787_s_at | ECT2 | 0.47 | 0.59 | GO:0032467;  GO:0005737;  GO:0005634;  GO:0030496 |  |
| 204603_at | EXO1 | 0.47 | 0.61 | GO:0005737;  GO:0005654 |  |
| 229538_s_at | IQGAP3 | 0.46 | 0.51 |  |  |
| 223307_at | CDCA3 | 0.46 | 0.60 |  |  |
| 218796_at | FERMT1 | 0.45 | 0.56 |  |  |
| 202580_x_at | FOXM1 | 0.45 | 0.70 | GO:0005737;  GO:0005634;  GO:0005654 |  |
| 218039_at | NUSAP1 | 0.45 | 0.61 | GO:0000281;  GO:0005737 |  |
| 220638_s_at | CBLC | 0.45 | 0.59 | GO:0005634;  GO:0061630 |  |
| 205429_s_at | MPP6 | 0.44 | 0.62 |  |  |
| 229610_at | CKAP2L | 0.44 | 0.51 |  |  |
| 224839_s_at | GPT2 | 0.44 | 0.41 |  |  |
| 210115_at | RPL39L | 0.43 | 0.54 |  |  |
| 220840_s_at | C1orf112 | 0.43 | 0.46 |  |  |
| 219874_at | SLC12A8 | 0.42 | 0.47 |  |  |
| 203700_s_at | DIO2 | 0.42 | 0.51 |  |  |
| 227350_at | HELLS | 0.41 | 0.45 | GO:0005634;  GO:0005524 |  |
| 218883_s_at | CENPU | 0.41 | 0.47 | GO:0005737;  GO:0005634 |  |
| 226287_at | CCDC34 | 0.40 | 0.50 |  |  |
| 232065_x_at | CENPL | 0.39 | 0.42 |  |  |
| 209434_s_at | PPAT | 0.37 | 0.39 |  |  |
| 227103_s_at | ECE2 | 0.37 | 0.58 | GO:0004222 |  |
| 239148_at | MARVELD3 | 0.36 | 0.43 |  |  |
| 205247_at | NOTCH4 | -0.33 | -0.36 | GO:0005509; GO:0005887; GO:0016021; GO:0005576; GO:0005886 |  |
| 59375_at | MYO15B | -0.33 | -0.39 |  |  |
| 205752_s_at | GSTM5 | -0.34 | -0.38 |  |  |
| 228127_at | KCNK3 | -0.34 | -0.37 | GO:0016021;  GO:0005886;  GO:0005887;  GO:0016021;  GO:0005887 |  |
| 212975_at | DENND3 | -0.34 | -0.37 |  |  |
| 226673_at | SH2D3C | -0.34 | -0.35 | GO0007125;  GO:0043547 |  |
| 230595_at | PGM5-AS1 | -0.34 | -0.41 |  |  |
| 227780_s_at | ECSCR | -0.34 | -0.39 | GO:0043547;  GO:0001525;  GO:0016021;  GO:0005886 |  |
| 219167_at | RASL12 | -0.35 | -0.42 |  |  |
| 1552388_at | FLJ30901  ///SCUBE1 | -0.35 | -0.42 |  |  |
| 212494_at | TNS2 | -0.35 | -0.44 | GO:0008285;  GO:0005886;  GO:0035556 |  |
| 227923_at | SHANK3 | -0.35 | -0.41 | GO:0005886 |  |
| 213103_at | STARD13 | -0.36 | -0.46 | GO0007125;  GO:0043547 |  |
| 228728_at | CPED1 | -0.36 | -0.47 |  |  |
| 228807_at | ASPA | -0.36 | -0.38 | GO:0070062 |  |
| 211416_x_at | GGTLC1 | -0.36 | -0.43 |  |  |
| 225369_at | ESAM | -0.36 | -0.43 | GO:0016021;  GO:0005886;  GO:0070062 |  |
| 201497_x_at | MYH11 | -0.36 | -0.48 | GO:0070062 |  |
| 40560_at | TBX2 | -0.37 | -0.50 |  |  |
| 203766_s_at | LMOD1 | -0.37 | -0.44 |  |  |
| 217897_at | FXYD6 | -0.37 | -0.37 | GO:0016021; GO:0005886 |  |
| 207317_s_at | CASQ2 | -0.37 | -0.39 | GO:0005509 |  |
| 205382_s_at | CFD | -0.37 | -0.47 | GO:0006508; GO:0070062;  GO:0005615 |  |
| 215349_at | BTBD18 | -0.38 | -0.38 |  |  |
| 214770_at | MSR1 | -0.38 | -0.57 | GO:0005887; GO:0016021; GO:0005886 |  |
| 1554331_a_at | LRRC18 | -0.38 | -0.42 |  |  |
| 204368_at | SLCO2A1 | -0.38 | -0.44 | GO:0005215; GO:0005887; GO:0016021; GO:0005886 |  |
| 1560826_at | LOC101929398 | -0.38 | -0.40 |  |  |
| 204396_s_at | GRK5 | -0.38 | -0.45 | GO:0005886 |  |
| 205929_at | GPA33 | -0.38 | -0.50 | GO:0070062; GO:0005887 |  |
| 236118_at | GATA6-AS1 | -0.38 | -0.43 |  |  |
| 228875_at | FAM162B | -0.38 | -0.54 | GO:0016021 |  |
| 205700_at | HSD17B6 | -0.38 | -0.65 |  |  |
| 226950_at | ACVRL1 | -0.38 | -0.44 | GO0007125;  GO:0001525;  GO:0008285;  GO:0016021;  GO:0005886;  GO:0005887; |  |
| 231947_at | MYCT1 | -0.38 | -0.51 | GO:0016021 |  |
| 226985_at | FGD5 | -0.38 | -0.49 | GO:0043547 |  |
| 228665_at | CYYR1 | -0.38 | -0.42 | GO:0016021 |  |
| 220765_s_at | LIMS2 | -0.39 | -0.37 | GO:0005886 |  |
| 204343_at | ABCA3 | -0.39 | -0.54 | GO:0005215; GO:0016887; GO:0016021; GO:0005886;  GO:0005615 |  |
| 1555216_a_at | LOC101060604  ///SLC7A5P2  ///SLC7A5P1 | -0.39 | -0.40 |  |  |
| 1563629_a_at | ERVK13-1 | -0.39 | -0.39 | GO:0016021; GO:0005886 |  |
| 219213_at | JAM2 | -0.39 | -0.45 | GO:0016021;  GO:0005886;  GO:0005887 |  |
| 206481_s_at | LDB2 | -0.39 | -0.49 |  |  |
| 228618_at | PEAR1 | -0.39 | -0.50 | GO:0016021 |  |
| 220006_at | EFCC1 | -0.39 | -0.42 | GO:0005509 |  |
| 226056_at | ARHGAP31 | -0.39 | -0.44 | GO0007125;  GO:0043547 |  |
| 221667_s_at | HSPB8 | -0.39 | -0.40 |  |  |
| 205934_at | PLCL1 | -0.39 | -0.46 | GO:0005886;  GO:0004871;  GO:0035556 |  |
| 226101_at | PRKCE | -0.39 | -0.37 | GO:0004871; GO:0007155; GO:0035556; GO:0005886 |  |
| 203865_s_at | ADARB1 | -0.39 | -0.63 | GO:0008285 |  |
| 229893_at | FRMD3 | -0.40 | -0.54 | GO:0016021 |  |
| 227618_at | NHSL2 | -0.40 | -0.49 |  |  |
| 204442_x_at | LTBP4 | -0.40 | -0.39 | GO:0005576;  GO:0005615;  GO:0005509;  GO:0070062 |  |
| 208510_s_at | PPARG | -0.40 | -0.52 | GO0007125 |  |
| 235849_at | SCARA5 | -0.40 | -0.62 | GO:0005887; |  |
| 229172_at | HSPA12B | -0.40 | -0.43 |  |  |
| 203088_at | FBLN5 | -0.40 | -0.48 | GO:0005576;  GO:0005615;  GO:0005509;  GO:0070062 |  |
| 210155_at | MYOC | -0.40 | -0.43 | GO:0005615;  GO:0070062 |  |
| 219719_at | HIGD1B | -0.40 | -0.52 | GO:0016021 |  |
| 1553448_at | FLJ34503 | -0.40 | -0.40 |  |  |
| 220570_at | RETN | -0.40 | -0.44 | GO:0005615;  GO:0070062 |  |
| 204042_at | WASF3 | -0.41 | -0.48 | GO:0070062 |  |
| 244655_at | LOC100507311 | -0.41 | -0.46 |  |  |
| 232686_at | SIGLEC17P | -0.41 | -0.39 |  |  |
| 205507_at | ARHGEF15 | -0.41 | -0.44 | GO:0043547 |  |
| 236079_at | LOC202025 | -0.41 | -0.49 |  |  |
| 238197_at | GATA5 | -0.41 | -0.47 |  |  |
| 210619_s_at | HYAL1 | -0.41 | -0.56 | GO:0005615;  GO:0070062 |  |
| 221204_s_at | CRTAC1 | -0.41 | -0.47 | GO:0005509;  GO:0070062 |  |
| 204428_s_at | LCAT | -0.41 | -0.45 | GO:0016021;  GO:0005576;  GO:0005615;  GO:0070062 |  |
| 1559605_a_at | LOC285043 | -0.41 | -0.50 |  |  |
| 232203_at | NKD1 | -0.41 | -0.48 | GO:0005886;  GO:0005509 |  |
| 203812_at | SLIT3 | -0.42 | -0.42 | GO:0008285;  GO:0005576;  GO:0005615;  GO:0005509;  GO:0008201;  GO:0005576 |  |
| 1556364_at | ADAMTS9-AS2 | -0.42 | -0.57 |  |  |
| 204468_s_at | TIE1 | -0.42 | -0.48 | GO0007125;  GO:0001525;  GO:0016021;  GO:0005887 |  |
| 223749_at | C1QTNF2 | -0.42 | -0.41 | GO:0005615 |  |
| 223600_s_at | KIAA1683 | -0.42 | -0.41 |  |  |
| 209897_s_at | SLIT2 | -0.42 | -0.53 | GO:0005886;  GO:0005576;  GO:0005615;  GO:0005509;  GO:0008201;  GO:0070062;  GO:0005576 |  |
| 219478_at | WFDC1 | -0.42 | -0.52 | GO:0005615 |  |
| 228554_at | PGR | -0.42 | -0.59 | GO0007125 |  |
| 226380_at | PTPN21 | -0.42 | -0.70 |  |  |
| 240856_at | FFAR4 | -0.42 | -0.49 | GO:0016021;  GO:0005886;  GO:0005887 |  |
| 203549_s_at | LPL | -0.42 | -0.60 | GO:0070062; GO:0008201; GO:0005886;  GO:0005615 |  |
| 213247_at | SVEP1 | -0.42 | -0.41 | GO:0007155;  GO:0005576;  GO:0005509 |  |
| 227775_at | CELF6 | -0.43 | -0.49 |  |  |
| 206762_at | KCNA5 | -0.43 | -0.45 | GO:0005886;  GO:0005887 |  |
| 206979_at | C8B | -0.43 | -0.45 | GO:0005576;  GO:0005615;  GO:0070062 |  |
| 238376_at | LOC100505564 | -0.43 | -0.46 |  |  |
| 215184_at | DAPK2 | -0.43 | -0.53 | GO:0016021;  GO:0035556 |  |
| 204072_s_at | FRY | -0.43 | -0.66 |  |  |
| 204365_s_at | REEP1 | -0.43 | -0.48 | GO:0016021 |  |
| 225915_at | CAB39L | -0.43 | -0.62 | GO:0070062 |  |
| 219091_s_at | MMRN2 | -0.43 | -0.51 | GO:0001525;  GO:0005615;  GO:0070062 |  |
| 218820_at | C14orf132 | -0.43 | -0.49 | GO:0016021 |  |
| 222161_at | NAALAD2 | -0.43 | -0.53 | GO:0016021;  GO:0005886;  GO:0006508 |  |
| 242809_at | IL1RL1 | -0.43 | -0.44 | GO0007125;  GO:0016021;  GO:0005886 |  |
| 204793_at | GPRASP1 | -0.43 | -0.42 |  |  |
| 1556314_a_at | ZNF366 | -0.44 | -0.47 |  |  |
| 220150_s_at | FAM184A | -0.44 | -0.55 | GO:0005615 |  |
| 213664_at | SLC1A1 | -0.44 | -0.64 | GO:0016021;  GO:0005886;  GO:0005887;  GO:0070062 |  |
| 226397_s_at | PHACTR1 | -0.44 | -0.46 |  |  |
| 227480_at | SUSD2 | -0.44 | -0.82 | GO:0016021;  GO:0005886;  GO:0070062 |  |
| 209894_at | LEPROT///LEPR | -0.44 | -0.58 |  |  |
| 202222_s_at | DES | -0.44 | -0.42 | GO:0070062 |  |
| 241742_at | PRAM1 | -0.44 | -0.38 |  |  |
| 213316_at | KIAA1462 | -0.44 | -0.56 | GO:0007155 |  |
| 225207_at | PDK4 | -0.44 | -0.71 |  |  |
| 1553447_at | AGBL1 | -0.44 | -0.47 | GO:0006508 |  |
| 222885_at | EMCN | -0.44 | -0.60 | GO:0001525;  GO:0016021;  GO:0005886;  GO:0005576 |  |
| 203571_s_at | ADIRF | -0.44 | -0.51 | GO:0070062 |  |
| 1558569_at | LOC100131541 | -0.44 | -0.49 |  |  |
| 226028_at | ROBO4 | -0.45 | -0.58 | GO:0001525;  GO:0016021;  GO:0070062 |  |
| 219568_x_at | SOX18 | -0.45 | -0.51 | GO:0001525 |  |
| 1552553_a_at | NLRC4 | -0.45 | -0.43 |  |  |
| 230661_at | LOC286191 | -0.45 | -0.43 |  |  |
| 220027_s_at | RASIP1 | -0.45 | -0.49 | GO0007125;  GO:0001525 |  |
| 230132_at | PCAT19 | -0.45 | -0.51 |  |  |
| 215318_at | MINOS1P1 | -0.45 | -0.56 |  |  |
| 221276_s_at | SYNC | -0.45 | -0.51 |  |  |
| 202524_s_at | SPOCK2 | -0.45 | -0.38 | GO0007125;  GO:0005509 |  |
| 233326_at | LOC101928882  ///CCDC39 | -0.45 | -0.49 |  |  |
| 210549_s_at | CCL23 | -0.45 | -0.55 | GO0007125;  GO:0043547;  GO:0008285;  GO:0005576;  GO:0005615;  GO:0008201 |  |
| 219064_at | ITIH5 | -0.45 | -0.67 | GO:0005576 |  |
| 204677_at | CDH5 | -0.45 | -0.55 | GO:0008285;  GO:0007155;  GO:0016021;  GO:0005886;  GO:0005509 |  |
| 219820_at | SLC6A16 | -0.45 | -0.44 | GO:0016021;  GO:0005887 |  |
| 239770_at | ESYT3 | -0.46 | -0.80 | GO:0005887; GO:0016021 |  |
| 241299_at | LOC102724611 | -0.46 | -0.51 |  |  |
| 218876_at | TPPP3 | -0.46 | -0.45 | GO:0070062 |  |
| 222925_at | DCDC2 | -0.46 | -0.67 | GO:0035556 |  |
| 205608_s_at | ANGPT1 | -0.46 | -0.53 | GO:0043547;  GO:0001525;  GO:0005886;  GO:0005576;  GO:0005615;  GO:0005088;  GO:0070062 |  |
| 220560_at | C11orf21 | -0.46 | -0.46 |  |  |
| 219957_at | RUFY2 | -0.46 | -0.57 |  |  |
| 226304_at | HSPB6 | -0.46 | -0.48 |  |  |
| 219958_at | TMEM74B | -0.46 | -0.45 | GO:0016021 |  |
| 210168_at | C6 | -0.46 | -0.52 | GO:0005576;  GO:0070062 |  |
| 235306_at | GIMAP8 | -0.46 | -0.51 |  |  |
| 213541_s_at | ERG | -0.47 | -0.50 | GO0007125;  GO:0004871 |  |
| 220736_at | SLC19A3 | -0.47 | -0.51 | GO:0016021;  GO:0005886;  GO:0005887 |  |
| 205496_at | KIAA0408 | -0.47 | -0.48 |  |  |
| 235183_at | FILIP1 | -0.47 | -0.85 |  |  |
| 229510_at | MS4A14 | -0.47 | -0.43 | GO:0016021 |  |
| 244276_at | KLB | -0.47 | -0.56 | GO:0005509;  GO:0004871;  GO:0043547;  GO:0005088 |  |
| 214945_at | LOC101930363  ///LOC101928349  ///LOC100507387  ///FAM153C  ///FAM153A  ///FAM153B | -0.47 | -0.48 |  |  |
| 219059_s_at | LYVE1 | -0.47 | -0.52 | GO0007125;  GO:0007155;  GO:0016021;  GO:0005886;  GO:0005887;  GO:0070062 |  |
| 240189_at | ACOXL | -0.47 | -0.63 |  |  |
| 226992_at | NOSTRIN | -0.48 | -0.87 | GO0007125;  GO:0005886 |  |
| 208109_s_at | LINC00597 | -0.48 | -0.64 |  |  |
| 229687_s_at | PRDM11 | -0.48 | -0.44 |  |  |
| 201540_at | FHL1 | -0.48 | -0.59 | GO:0005886; |  |
| 226856_at | TMEM110-  MUSTN1///  MUSTN1///  TMEM110 | -0.48 | -0.59 |  |  |
| 228268_at | FMO2 | -0.48 | -0.57 | GO:0016021 |  |
| 207069_s_at | SMAD6 | -0.48 | -0.64 | GO:0008285;  GO:0016021 |  |
| 242541_at | ABCA9 | -0.48 | -0.46 | GO:0016021;  GO:0005215;  GO:0016887 |  |
| 1556711_at | FAM216B | -0.48 | -0.52 |  |  |
| 205632_s_at | PIP5K1B | -0.48 | -0.62 |  |  |
| 230250_at | PTPRB | -0.48 | -0.65 | GO:0001525;  GO:0005887 |  |
| 227419_x_at | PLAC9 | -0.49 | -0.59 | GO:0005576 |  |
| 1562921_at | EP300-AS1 | -0.49 | -0.52 |  |  |
| 235666_at | ITGA8 | -0.49 | -0.61 | GO:0007155;  GO:0005886; |  |
| 221035_s_at | TEX14 | -0.49 | -0.47 | GO:0070062 |  |
| 226071_at | ADAMTSL4 | -0.49 | -0.47 | GO:0006508 |  |
| 210944_s_at | CAPN3 | -0.49 | -0.60 | GO0007125;  GO:0005886;  GO:0005509;  GO:0004871;  GO:0006508 |  |
| 205935_at | FOXF1 | -0.49 | -0.57 |  |  |
| 205392_s_at | CCL15-CCL14  ///CCL14 | -0.49 | -0.54 |  |  |
| 209993_at | ABCB1 | -0.49 | -0.54 | GO:0016021;  GO:0005886;  GO:0005215;  GO:0070062 |  |
| 244422_at | LOC101928370 | -0.49 | -0.44 |  |  |
| 228731_at | GUCY1A2 | -0.49 | -0.68 | GO:0035556; GO:0005886 |  |
| 236320_at | CCDC17 | -0.49 | -0.49 |  |  |
| 228232_s_at | VSIG2 | -0.49 | -0.60 | GO:0005887 |  |
| 236396_at | LOC101927263 | -0.49 | -0.55 |  |  |
| 212713_at | MFAP4 | -0.50 | -0.58 | GO:0007155;  GO:0005576;  GO:0070062 |  |
| 213706_at | GPD1 | -0.50 | -0.54 | GO:0070062 |  |
| 222717_at | SDPR | -0.50 | -0.55 |  |  |
| 229222_at | ACSS3 | -0.50 | -0.58 |  |  |
| 231991_at | CCM2L | -0.50 | -0.60 |  |  |
| 223597_at | ITLN1 | -0.50 | -0.54 | GO:0070062 |  |
| 225575_at | LIFR | -0.50 | -0.56 | GO:0016021;  GO:0070062  GO:0005886;  GO:0005887 |  |
| 219949_at | LRRC2 | -0.50 | -0.51 |  |  |
| 229584_at | LRRK2 | -0.50 | -0.61 | GO:0043547;  GO:0005886;  GO:0005615;  GO:0035556;  GO:0070062 |  |
| 1569608_x_at | LOC105379426 | -0.50 | -0.58 |  |  |
| 214053_at | ERBB4 | -0.51 | -0.65 | GO0007125;  GO:0043547;  GO:0008285;  GO:0016021;  GO:0005886;  GO:0005576;  GO:0005088 |  |
| 219452_at | DPEP2 | -0.51 | -0.52 | GO:0006508 |  |
| 216333_x_at | TNXB///TNXA | -0.51 | -0.57 |  |  |
| 211696_x_at | HBB | -0.51 | -0.42 | GO:0016021;  GO:0005576;  GO:0070062 |  |
| 228770_at | GPR146 | -0.51 | -0.52 | GO:0016021;  GO:0005886 |  |
| 220244_at | LINC00312 | -0.51 | -0.52 |  |  |
| 233305_at | NECAB1 | -0.51 | -0.52 | GO:0005509 |  |
| 205819_at | MARCO | -0.51 | -0.56 | GO:0016021;  GO:0005886;  GO:0005887; |  |
| 226625_at | TGFBR3 | -0.51 | -0.39 | GO:0035556; GO:0070062; GO:0008201; GO:0005576; GO:0005887; GO:0016021; GO:0005886;  GO:0005615 |  |
| 229824_at | SHC3 | -0.51 | -0.68 | GO0007125;  GO:0043547;  GO:0005886;  GO:0005088;  GO:0004871 |  |
| 1557136_at | ATP13A4 | -0.52 | -0.54 | GO:0016021;  GO:0005886;  GO:0005887;  GO:0016887 |  |
| 220156_at | EFCAB1 | -0.52 | -0.44 |  |  |
| 233002_at | PPP4R4 | -0.52 | -0.54 |  |  |
| 227108_at | STARD9 | -0.52 | -0.64 | GO:0016887 |  |
| 227148_at | PLEKHH2 | -0.52 | -0.56 | GO:0005886 |  |
| 241986_at | BMPER | -0.52 | -0.77 | GO:0005576;  GO:0005615 |  |
| 228335_at | CLDN11 | -0.52 | -0.64 | GO:0016021;  GO:0005886;  GO:0070062 |  |
| 206167_s_at | ARHGAP6 | -0.52 | -0.77 | GO0007125;  GO:0043547 |  |
| 204073_s_at | MYRF | -0.52 | -0.64 | GO:0016021;  GO:0006508 |  |
| 202992_at | C7 | -0.53 | -0.59 | GO:0070062; GO:0005576 |  |
| 232473_at | LOC101928524 | -0.53 | -0.53 |  |  |
| 213715_s_at | KANK3 | -0.53 | -0.57 |  |  |
| 207195_at | CNTN6 | -0.53 | -0.66 | GO:0007155;  GO:0016021;  GO:0005886 |  |
| 236304_at | LINC00702 | -0.53 | -0.58 |  |  |
| 231773_at | ANGPTL1 | -0.53 | -0.58 | GO:0005615;  GO:0070062 |  |
| 239229_at | PHEX | -0.53 | -0.55 | GO:0016021;  GO:0005886;  GO:0005887;  GO:0006508 |  |
| 229012_at | C9orf24 | -0.53 | -0.52 |  |  |
| 228737_at | TOX2 | -0.54 | -0.41 |  |  |
| 209763_at | CHRDL1 | -0.54 | -0.85 | GO:0005576 |  |
| 228739_at | CYS1 | -0.54 | -0.65 | GO:0070062 |  |
| 1560169_at | LOC101927069 | -0.54 | -0.61 |  |  |
| 1554503_a_at | OSCAR | -0.54 | -0.55 | GO:0016021;  GO:0005886;  GO:0005576;  GO:0070062 |  |
| 208195_at | TTN | -0.54 | -0.54 | GO:0005576;  GO:0005509;  GO:0070062 |  |
| 1552398_a_at | CLEC12A | -0.54 | -0.57 | GO:0016021;  GO:0005886 |  |
| 230472_at | IRX1 | -0.55 | -0.50 |  |  |
| 229019_at | ZNF385B | -0.55 | -0.75 |  |  |
| 1558795_at | LOC101928307 | -0.55 | -0.58 |  |  |
| 230624_at | SLC25A27 | -0.55 | -0.79 | GO:0016021 |  |
| 228692_at | PREX2 | -0.55 | -0.83 | GO:0043547;  GO:0005886;  GO:0035556 |  |
| 227929_at | LIN7A | -0.56 | -0.57 | GO:0005886;  GO:0070062 |  |
| 233527_at | LOC101929340 | -0.56 | -0.72 |  |  |
| 210004_at | OLR1 | -0.56 | -0.65 | GO:0007155;  GO:0016021;  GO:0005886;  GO:0005887;  GO:0006508;  GO:0070062 |  |
| 232224_at | MASP1 | -0.56 | -0.56 | GO:0005576;  GO:0005615;  GO:0005509;  GO:0006508 |  |
| 219689_at | SEMA3G | -0.56 | -0.48 | GO:0005615;  GO:0070062 |  |
| 226303_at | PGM5 | -0.57 | -0.85 | GO:0007155 |  |
| 235334_at | ST6GALNAC3 | -0.57 | -0.53 | GO:0016021 |  |
| 1557359_at | LINC01268 | -0.57 | -0.60 |  |  |
| 238332_at | ANKRD29 | -0.57 | -0.71 | GO:0016021 |  |
| 211734_s_at | FCER1A | -0.57 | -0.67 | GO:0016021;  GO:0005886;  GO:0005887; |  |
| 207496_at | MS4A2 | -0.57 | -0.50 | GO:0005886;  GO:0005887 |  |
| 242714_at | LOC101928429 | -0.57 | -0.63 |  |  |
| 229339_at | MYOCD | -0.57 | -0.61 | GO:0008285 |  |
| 235568_at | MCEMP1 | -0.57 | -0.64 | GO:0016021 |  |
| 222073_at | COL4A3 | -0.57 | -1.16 | GO:0008285;  GO:0007155;  GO:0005576 |  |
| 228568_at | MYZAP | -0.58 | -0.70 | GO:0035556 |  |
| 243802_at | DNAH12 | -0.58 | -0.64 | GO:0016887 |  |
| 217504_at | ABCA6 | -0.58 | -0.63 | GO:0016021;  GO:0005215  GO:0016887 |  |
| 220133_at | ODAM | -0.58 | -0.60 | GO:0043547;  GO:0005576;  GO:0005615 |  |
| 204154_at | CDO1 | -0.58 | -0.73 |  |  |
| 210550_s_at | RASGRF1 | -0.58 | -0.65 | GO0007125;  GO:0043547;  GO:0005886;  GO:0005088;  GO:0035556 |  |
| 206029_at | ANKRD1 | -0.58 | -0.58 |  |  |
| 220110_s_at | NXF3 | -0.58 | -0.47 |  |  |
| 224061_at | INMT | -0.59 | -0.78 |  |  |
| 209789_at | CORO2B | -0.59 | -0.61 |  |  |
| 237281_at | AKAP14 | -0.59 | -0.64 |  |  |
| 206208_at | CA4 | -0.60 | -0.63 | GO:0016021;  GO:0005886;  GO:0070062 |  |
| 213900_at | FAM189A2 | -0.60 | -0.71 | GO:0016021 |  |
| 228698_at | SOX7 | -0.60 | -0.49 | GO:0008285 |  |
| 229116_at | CNKSR2 | -0.60 | -0.53 | GO:0005886;  GO:0070062 |  |
| 231084_at | CFAP43 | -0.60 | -0.68 |  |  |
| 241310_at | NEK5 | -0.60 | -0.74 |  |  |
| 38691_s_at | SFTPC | -0.61 | -0.69 | GO:0016021;  GO:0005576;  GO:0005615 |  |
| 219140_s_at | RBP4 | -0.61 | -0.77 | GO:0005576;  GO:0005615;  GO:0005215;  GO:0070062 |  |
| 213240_s_at | KRT4 | -0.61 | -0.59 |  |  |
| 229302_at | TMEM178A | -0.61 | -0.63 | GO:0016021 |  |
| 229781_at | LOC100506725 | -0.62 | -0.66 |  |  |
| 239477_at | EFHB | -0.62 | -0.71 | GO:0005509 |  |
| 219993_at | SOX17 | -0.62 | -0.63 | GO:0001525 |  |
| 244455_at | KCNT2 | -0.62 | -0.83 | GO:0016021 |  |
| 205554_s_at | DNASE1L3 | -0.62 | -0.55 | GO:0005576;  GO:0005509 |  |
| 205200_at | EXOSC7  ///CLEC3B | -0.62 | -0.73 |  |  |
| 238062_at | GPIHBP1 | -0.62 | -0.68 |  |  |
| 207547_s_at | FAM107A | -0.63 | -0.60 |  |  |
| 206007_at | PRG4 | -0.63 | -0.80 | GO:0005576; |  |
| 210258_at | RGS13 | -0.63 | -0.57 | GO:0043547;  GO:0005886 |  |
| 204579_at | FGFR4 | -0.63 | -0.79 | GO:0043547;  GO:0016021;  GO:0005886;  GO:0005887;  GO:0005576;  GO:0005088;  GO:0008201 |  |
| 238116_at | DYNLRB2 | -0.64 | -0.83 | GO:0070062 |  |
| 205638_at | ADGRB3 | -0.64 | -0.74 | GO:0043547;  GO:0016021;  GO:0005886 |  |
| 205612_at | MMRN1 | -0.64 | -0.69 | GO:0007155;  GO:0005576;  GO:0005509 |  |
| 227848_at | PEBP4 | -0.64 | -0.86 | GO:0016021;  GO:0070062 |  |
| 220380_at | DNASE2B | -0.64 | -0.55 | GO:0005576 |  |
| 205883_at | ZBTB16 | -0.64 | -0.61 | GO:0008285;  GO:0005886 |  |
| 229973_at | ERICH3 | -0.65 | -0.68 |  |  |
| 220269_at | ZBBX | -0.65 | -0.72 |  |  |
| 210078_s_at | KCNAB1 | -0.65 | -0.62 | GO:0016021;  GO:0005886; |  |
| 206488_s_at | CD36 | -0.65 | -0.66 | GO:0007155;  GO:0016021;  GO:0005886;  GO:0005887;  GO:0005615; |  |
| 1556026_at | LINC00893 | -0.65 | -0.62 |  |  |
| 230670_at | IGSF10 | -0.65 | -0.44 | GO:0005576 |  |
| 220003_at | LRRC36 | -0.66 | -0.70 |  |  |
| 210390_s_at | CCL15-CCL14  ///CCL15 | -0.66 | -0.70 |  |  |
| 1560850_at | LOC101926959 | -0.67 | -0.96 |  |  |
| 205978_at | KL | -0.67 | -0.89 | GO:0043547;  GO:0016021;  GO:0005886;  GO:0005887;  GO:0005576;  GO:0005615;  GO:0005088;  GO:0004871;  GO:0070062 |  |
| 235670_at | STX11 | -0.67 | -0.71 | GO:0016021;  GO:0005886 |  |
| 242496_at | ART4 | -0.67 | -0.72 | GO:0016021;  GO:0005886 |  |
| 233591_at | LOC101930164 | -0.67 | -0.70 |  |  |
| 206702_at | TEK | -0.67 | -0.73 | GO0007125;  GO:0043547;  GO:0001525;  GO:0005886;  GO:0005887;  GO:0005576;  GO:0005088 |  |
| 235210_s_at | SBSPON | -0.68 | -0.49 |  |  |
| 229641_at | CCBE1 | -0.68 | -0.82 | GO:0005615;  GO:0005509 |  |
| 206068_s_at | ACADL | -0.69 | -0.78 |  |  |
| 222722_at | OGN | -0.69 | -1.00 | GO:0005576;  GO:0005615;  GO:0008201;  GO:0070062 |  |
| 230964_at | FREM2 | -0.69 | -0.78 | GO:0007155;  GO:0016021;  GO:0005886;  GO:0070062 |  |
| 209841_s_at | LRRN3 | -0.69 | -0.85 | GO:0016021 |  |
| 213974_at | ADAMTSL3 | -0.69 | -0.84 | GO:0006508 |  |
| 206658_at | UPK3B | -0.70 | -0.62 | GO:0016021;  GO:0005886;  GO:0070062 |  |
| 238222_at | GKN2 | -0.71 | -0.93 | GO:0005615 |  |
| 228915_at | DACH1 | -0.71 | -0.77 |  |  |
| 229542_at | C20orf85 | -0.71 | -0.73 |  |  |
| 205866_at | FCN3 | -0.71 | -0.70 | GO:0005576;  GO:0006508 |  |
| 204931_at | TCF21 | -0.72 | -0.86 |  |  |
| 220170_at | FHL5 | -0.72 | -0.89 |  |  |
| 210383_at | SCN1A | -0.72 | -0.85 | GO:0016021;  GO:0005886 |  |
| 235771_at | LINC00472 | -0.73 | -0.73 |  |  |
| 1559277_at | FLJ35700 | -0.73 | -0.86 |  |  |
| 228434_at | BTNL9 | -0.73 | -0.89 | GO:0016021 |  |
| 238720_at | LOC101927057  ///OMG | -0.74 | -0.83 |  |  |
| 239349_at | C1QTNF7 | -0.74 | -1.08 | GO:0005576 |  |
| 1553645_at | CCDC141 | -0.76 | -0.77 |  |  |
| 232122_s_at | VEPH1 | -0.76 | -0.94 | GO:0005886 |  |
| 235649_at | ADAMTS8 | -0.77 | -0.84 | GO:0008285;  GO:0008201;  GO:0006508 |  |
| 228885_at | MAMDC2 | -0.78 | -1.21 |  |  |
| 239150_at | SNTN | -0.78 | -0.92 |  |  |
| 207302_at | SGCG | -0.79 | -0.63 | GO:0016021;  GO:0005886 |  |
| 230720_at | RNF182 | -0.79 | -0.94 | GO:0016021 |  |
| 211276_at | TCEAL2 | -0.79 | -0.72 |  |  |
| 220351_at | ACKR4 | -0.80 | -0.90 | GO:0005886;  GO:0005887; |  |
| 207519_at | SLC6A4 | -0.81 | -0.80 | GO:0016021;  GO:0005886;  GO:0005887 |  |
| 204719_at | ABCA8 | -0.81 | -1.16 | GO:0016021;  GO:0005886;  GO:0005215;  GO:0016887 |  |
| 226228_at | AQP4 | -0.81 | -1.06 | GO:0016021;  GO:0005886;  GO:0005887;  GO:0005215 |  |
| 209612_s_at | ADH1B | -0.81 | -1.14 |  |  |
| 219937_at | TRHDE | -0.82 | -0.80 | GO0007125;  GO:0005887;  GO:0006508;  GO:0070062 |  |
| 243813_at | LINC00968 | -0.82 | -0.86 |  |  |
| 213317_at | CLIC5 | -0.82 | -1.12 | GO:0070062 |  |
| 223836_at | FGFBP2 | -0.83 | -0.65 | GO:0005615 |  |
| 223623_at | C2orf40 | -0.84 | -0.99 | GO:0005615 |  |
| 238018_at | FAM150B | -0.84 | -0.87 | GO:0005576 |  |
| 236029_at | FAT3 | -0.84 | -0.96 | GO:0016021;  GO:0005886;  GO:0005509 |  |
| 1564194_a_at | MS4A15 | -0.86 | -0.91 | GO:0016021 |  |
| 219230_at | TMEM100 | -0.87 | -1.05 | GO:0001525;  GO:0016021;  GO:0005886 |  |
| 206742_at | PIR-FIGF///FIGF | -0.88 | -1.08 |  |  |
| 214135_at | CLDN18 | -0.88 | -1.13 | GO:0016021;  GO:0005886 |  |
| 230867_at | COL6A6 | -0.89 | -0.89 | GO:0007155;  GO:0005576 |  |
| 210096_at | CYP4B1 | -0.89 | -1.24 | GO:0016021 |  |
| 229151_at | SLC14A1 | -0.95 | -1.06 | GO:0016021;  GO:0005886;  GO:0005887 |  |
| 203980_at | FABP4 | -0.95 | -0.92 | GO:0005215; GO:0070062 |  |
| 1554012_at | RSPO2 | -0.96 | -0.98 | GO:0005576;  GO:0008201 |  |
| 227198_at | AFF3 | -0.96 | -1.09 |  |  |
| 206159_at | GDF10 | -0.97 | -1.12 | GO:0005615 |  |
| 210081_at | AGER | -0.97 | -1.02 | GO:0016021;  GO:0005886,  GO:0005887;  GO:0005576;  GO:0008201 |  |
| 241672_at | SERTM1 | -1.06 | -1.09 | GO:0016021 |  |
| 206651_s_at | CPB2 | -1.07 | -1.16 | GO:0005615;  GO:0006508;  GO:0070062 |  |
| 230469_at | RTKN2 | -1.08 | -1.13 | GO0007125 |  |
| 239650_at | NCKAP5 | -1.16 | -1.00 |  |  |
| 209470_s_at | GPM6A | -1.16 | -1.34 | GO:0016021;  GO:0005886;  GO:0070062 |  |
